# The Physician Himself

**Published:** 1889-09

**Authors:** 


THE PHYSICIAN HIMSELF and things that concern his reputation and success, by D. W. Cathell, M. D., Baltimore, Md. The ninth edition. Revised and enlarged. Philadelphia and London. F. A. Davis, Publisher, 1889. Cloth 291 large oc. pages. Price $2.00.

This is an admirable book full of sound advice and wise direction to the medical practitioner. It should be carefully observed at the outset of a professional career by every graduate in medicine. If its counsels be followed by the reader he cannot fail of success in life, not only as a practitioner, but as a man of good character and high standing in the community.

